# The Effectiveness of Bariatric Surgery on Treating Infertility in Women—A Systematic Review and Meta-Analysis

**DOI:** 10.3390/jcm13185569

**Published:** 2024-09-19

**Authors:** Hadeel Almutairi, Mohammad Sulaiman Aldhalea, Muhammad Abdulghani Almaaz, Sama Abdalaziz Aljuhani, Rena Ibrahim Aloraini, Abdulrahman Abdullah Alamoudi, Wajd Fahad Alkhalifah, Leen Ahmed Alrushaid, Haneen Wadi Alanzy, Meshal Alzuwayyid, Flora Abdulaziz Alrumaih, Moneerah Madeallah Al-harbi, Alaa Ahmad AL-Aboudi, Faisal Salem Alqadi, Reem Salem Alshammari

**Affiliations:** 1 Department of Surgery, College of Medicine, Qassim University, Buraydah 52571, Saudi Arabia; 411216313@qu.edu.sa (S.A.A.); 421202021@qu.edu.sa (R.I.A.); 412205560@qu.edu.sa (W.F.A.); 421202219@qu.edu.sa (L.A.A.); 411107568@qu.edu.sa (M.A.); 421202188@qu.edu.sa (F.A.A.); 412205537@qu.edu.sa (M.M.A.-h.); 411202044@qu.edu.sa (A.A.A.-A.); 2 Buraidah Central Hospital, Qassim Cluster, Ministry of Health, Buraydah 52367, Saudi Arabia; dr.mohamd@hotmail.com (M.S.A.); dr.maaz@yahoo.com (M.A.A.); 3 College of Medicine and Surgery, King Abdulaziz University, Jeddah 21589, Saudi Arabia; aalamoudi0338@stu.kau.edu.sa; 4 College of Medicine and Surgery, Northern Border University, Arar 73213, Saudi Arabia; st202101210@stu.nbu.edu.sa; 5 College of Medicine, King Khalid University, Abha 62521, Saudi Arabia; 439800341@kku.edu.sa; 6 College of Medicine, Northern Border University, Arar 73213, Saudi Arabia; st201801971@stu.nbu.edu.sa

**Keywords:** overactive bladder, prevalence, urinary incontinence, women’s health

## Abstract

**Background/Objectives:** Obesity is a growing global health concern, which increases the risk of various diseases and has seen a rising prevalence over time. The global prevalence of obesity among adults has doubled over time. Obesity significantly impacts health by increasing the risk of a range of severe medical conditions. Cardiovascular diseases, such as heart attacks and strokes, are more prevalent in individuals with obesity due to factors like high blood pressure and abnormal cholesterol levels. This systematic review and meta-analysis sought to establish the effectiveness of bariatric surgery in treating infertility in women. **Methods:** This systematic review and meta-analysis were conducted in accordance with the Preferred Reporting Items for Systematic Review and Meta-Analysis (PRISMA) guidelines. A broad electronic search was conducted through PubMed, Web Science, and Medline databases for studies published between April 2017 and October 2023. The search strategy used the following terms: bariatric surgery, metabolic surgery, bariatric surgical procedures, stomach stapling, infertility, and fertility issues. The data were analyzed using the Revman version 5.1.2 software. **Results:** The results of the study show that despite the heterogeneity found in the studies, irregular menstrual cycles were found to reduce significantly in patients who underwent bariatric surgery (*p* = 0.01), with an RR of 0.22, at a 95% CI (0.06, 0.74). With regards to infertility, the results indicate that bariatric surgery reduced the level of infertility among the patients significantly (*p* = 0.00001), with an RR of 0.55, at a 95% CI (0.45, 0.68). Further, the results show bariatric surgery reduced rate of miscarriages among patients (*p* = 0.01), with an RR of 0.51, at a 95% CI (0.30, 0.86). Moreover, bariatric surgery reduced the level of congenital malfunction, but the effect was not statistically significant (*p* = 0.16), with an RR of 0.39, at a 95% CI (0.10, 1.45). However, the overall effect of bariatric surgery on treating infertility was found to be significantly effective (*p* = 0.0001), with an RR of 0.54, at a 95% CI (0.43, 0.68). This implies that bariatric surgery helps in weight loss, which improves ovulatory dysfunction and irregular menstruation while boosting spontaneous conception. **Conclusions:** This study found that bariatric surgery helps infertile women of a reproductive age to lose weight, which improves ovulatory dysfunction and irregular menstruation while boosting spontaneous conception. On the other hand, the study noted that after bariatric surgery, spontaneous conception can occur because of a decreased rate of miscarriage, increased fertility, reduced levels of congenital malfunction, and the restoration of regular menstrual cycles. Therefore, this study highlights the need to offer adequate preconception care and counselling to women who are about to be pregnant, both before and after bariatric surgery. Further, based on the fact that this study focused on general bariatric surgery, future research should focus on specific types of bariatric surgery to establish the most effective type of bariatric surgery in treating infertility in women.

## 1. Introduction

Obesity is a growing global health problem that increases the risk of numerous diseases like diabetes, hypertension, certain cancers, and respiratory conditions. The worldwide incidence of obesity in adults aged 18 and above has experienced a substantial rise, increasing from 25% in 1990 to 43% in 2022, representing a more than twofold increase over this time frame [1]. There are more than 890 million people who are obese, which makes up about 16% of the global adult population [1]. Individuals with obesity have a significantly higher likelihood of developing cardiovascular conditions such as atrial fibrillation, dyslipidemia, diabetes, and coronary artery disease [2]. Obesity is associated with a higher incidence of cardiovascular disorders due to factors such as obesity-related inflammation and visceral adiposity. These conditions contribute to the body’s impaired ability to utilize insulin efficiently and exacerbate the risk of developing serious health issues [3]. Obesity has been associated with infertility through various mechanisms, including those at the molecular level [4].

Women who are overweight or obese face a higher risk of miscarriage and stillbirth [5]. In women, obesity is significantly linked to a higher occurrence of menstrual irregularities [6]. Obesity can influence female fertility by altering oocyte development. Obese women have an altered follicular environment, including elevated insulin levels, lipids, and inflammatory markers, including lactate and the C-reactive protein [7]. Inflammatory pathways are critical for reproductive events such as follicle rupture during ovulation and trophoblast invasion into the receptive endometrium [4]. Therefore, the altered inflammatory environment in women who are obese is likely to affect these critical reproductive processes.

Obesity is a recognized risk factor for female infertility. Overweight and underweight women also face a heightened risk of reduced fertility. Data from 7327 pregnant women participating in the Collaborative Perinatal Project, conducted at 12 study centers across the United States, revealed that after adjusting for age, fecundability decreased for women who were underweight (fecundability OR = 0.94; 95% CI: 0.86, 1.03), overweight (fecundability OR = 0.84; 95% CI: 0.77, 0.92), and obese (fecundability OR = 0.72; 95% CI: 0.63, 0.83) compared to those with an optimal body mass index [8].

Weight loss results in better pregnancy and live birth rates for obese individuals with infertility [7]. Bariatric procedures are one of numerous treatments that may be utilized for weight loss, including lifestyle adjustments, dietary changes, increased physical activity, and medication [8]. Unlike general lifestyle modifications, bariatric surgery is indicated for specific cases, such as severe obesity or obesity-related health conditions. Some bariatric techniques involve significant alterations to the digestive tract, leading to malabsorption and substantial weight loss [8]. The Roux-en-Y gastric bypass (RYGB), sleeve gastrectomy, gastric banding (LAGB), the one-anastomosis gastric bypass (OAG), and the duodenal switch (BPD-DS) are among the primary bariatric surgical procedures that are used to reduce weight [9]. The techniques of these procedures vary; however, their shared goal is to reduce the size of the stomach and affect digestion to facilitate significant and long-term weight loss.

A thorough survey was conducted on 195 female patients undergoing bariatric surgery. The results showed that 71% of women who had anovulation before the surgery had a restoration of regular menstrual periods after the operation. Moreover, the restoration of regular menstrual function was strongly linked to a higher degree of weight loss attained with the surgical procedure [10].

Because there is not a lot of data available on how bariatric surgery affects reproductive health, we conducted a meta-analysis and systematic review to look into many essential factors. Firstly, we looked at how well bariatric surgery works to help obese women who are experiencing infertility and how surgery affects their capability to get pregnant again. We evaluated post-surgery outcomes for women, including blood testing and medical follow-ups. We aimed to give healthcare practitioners evidence-based recommendations on how effective bariatric surgery improves fertility outcomes for obese women. To ensure that clinical treatments are comprehensive and well informed, we considered implications on reproductive health, including how bariatric surgery impacts on fertility, safety, patient satisfaction, and recovery metrics.

In an area with limited and fragmented data, this study’s significance is its comprehensive examination of the impact of bariatric surgery on fertility outcomes. This meta-analysis is not the first on this topic; however, our research is uniquely positioned to address gaps in the literature by incorporating a broader range of studies, more stringent selection criteria, and a detailed analysis of various bariatric procedures and their specific effects on fertility.

In conclusion, with different effects depending on the particular operation, we hypothesize that bariatric surgery significantly improves fertility outcomes in obese women and that post-surgery weight loss correlates favorably with the restoration of regular menstrual cycles and elevated pregnancy rates.

## 2. Materials and Methods

### 2.1. Literature Search Strategy

The systematic review was conducted in accordance with the Preferred Reporting Items for Systematic Reviews and Meta-Analyses (PRISMA) guidelines and the Cochrane Handbook for Systematic Reviews. An extensive electronic search was performed using PubMed, Web Science, and Medline databases. The search was limited to studies published in English. The search terms used included combinations of keywords and medical subject headings (MeSH) relevant to the study objective. The literature search was conducted from 2017 to 2023. The search string was as follows: (bariatric surgery OR metabolic surgery OR bariatric surgical procedures OR stomach stapling) AND (infertility OR fertility issues) AND (management OR treatment) AND (females OR women OR effectiveness of bariatric surgery on treating infertility in females OR treatment of infertility in obese women OR effects of bariatric surgery on infertility in obese females). The inclusion criteria were studies published from 2017 to 2023 in English, with text available as abstracts. The types of articles included were books and documents, clinical trials, randomized controlled trials, review articles, and systematic reviews. All research considered was conducted on humans. Our review was limited to studies available through the selected electronic databases.

### 2.2. PROSPERO Registration

The protocol for this systematic review was retrospectively registered with the International Prospective Register of Systematic Reviews (PROSPERO) under the registration number [CRD42024558053].

### 2.3. Inclusion and Exclusion Criteria

The inclusion and exclusion criteria were defined based on the PICOS framework.

Population: Obese women (BMI ≥ 30 kg/m^2^) who have been infertile without a confirmed diagnosis of other primary or secondary causes of infertility. The search covered the literature from 1 April 2017 to 31 October 2023, using the following search string: bariatric surgery OR metabolic surgery OR bariatric surgical procedures OR stomach stapling AND infertility OR fertility issues AND management OR treatment AND females OR women OR effectiveness of bariatric surgery on treating infertility in females OR treatment of infertility in obese women OR effects of bariatric surgery on infertility in obese females.

Intervention: Bariatric surgery, including but not limited to a Roux-en-Y gastric bypass, sleeve gastrectomy, adjustable gastric banding, and biliopancreatic diversion with a duodenal switch.

Comparison: A comparison of fertility outcomes before and after undergoing bariatric surgery within the same cohort.

Outcomes: The primary outcomes evaluated were a reduction in infertility rates, miscarriage rates, and the restoration of regular menstrual cycles. Specifically, “miscarriage” was defined as the spontaneous loss of a pregnancy before the 20th week of gestation in the included studies. “Infertility” was defined as the inability to conceive after 12 months of regular, unprotected intercourse.

### 2.4. Study Design

Observational studies (cohort, case-control, and cross-sectional studies) and randomized controlled trials (RCTs) were also included—case reports, systematic reviews, meta-analyses, conference abstracts, and editorials were excluded.

### 2.5. Selection of Articles and Data Extraction

The selection process involved multiple stages to ensure the inclusion of relevant studies. Four independent reviewers (A.A.A., M.M.H., F.A.R., and L.A.R.) initially screened the titles and abstracts of articles retrieved from the search to identify potentially relevant studies. The full text of the selected articles was then reviewed independently by the same four reviewers to confirm eligibility based on the predefined inclusion and exclusion criteria. Data extraction was conducted separately by the four reviewers using a standardized form. The extracted data included study characteristics such as author(s), year of publication, country, study design, type of bariatric surgery, and sample size; patient characteristics such as age, social status, preoperative and postoperative BMI, and follow-up interval; and outcome measures such as indicators of fertility (hormonal levels, ovulation rates, pregnancy rates, and live birth rates) and postoperative follow-ups, including monitoring for any complications.

### 2.6. Study Quality

The Newcastle–Ottawa Quality Assessment Scale (NOS) was utilized to systematically evaluate the quality of the included studies by assigning up to one star for each numbered item in the selection and outcome categories, ensuring a comprehensive assessment of study robustness. To ensure the integrity of our dataset, we identified and removed duplicate articles using Mendeley Reference Manager: v2.71.0. This step was critical in preventing the duplication of data and maintaining the accuracy of our systematic review. Disagreements during the screening and data extraction phases were resolved through discussion and consensus among all reviewers.

### 2.7. Statistical Analysis

The data were analyzed using Revman version 5.1.2 software (The Cochrane Collaboration, London, UK). A random effect model was conducted, and the risk ratios (RRs) at 95% confidence intervals (CIs) were computed for categorical outcomes. The heterogeneity of the research was assessed using the Q test and the I° statistic, whereby they were classified into four quartiles: low, moderately low, moderately high, and high heterogeneity, ranging from 0% to <25%, 25% to <50%, >50 to <95, and >75%, respectively. Forest plots with 95% confidence intervals were used to display the data. A significance level of 0.05 was used for all analyses.

## 3. Results

**Figure 1:**
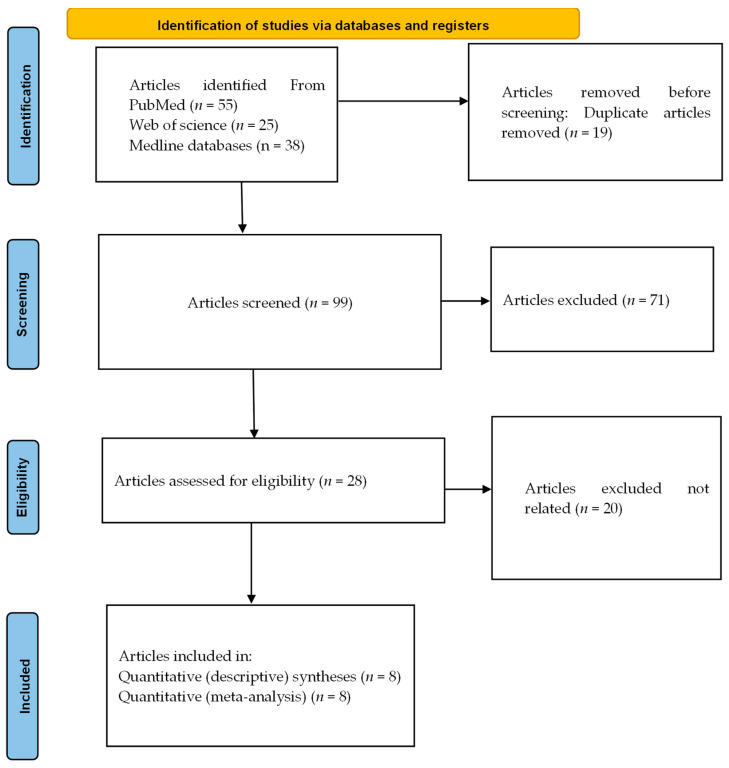
PRISMA flow diagram.

Figure 1 shows a PRISMA diagram of the studies that were included in this investigation. The search yielded 55 papers from PubMed, 25 from Medline, and 38 from Web of Science, totaling 118 articles. After removing 19 duplicates, 99 unique articles were included in the initial screening. In the screening phase, 71 articles were excluded, leaving 28 articles that were selected for eligibility assessment. In the eligibility phase, 20 articles were excluded since their contributions were irrelevant to the goals of the study. Finally, eight studies were included in systematic review and meta-analysis, and all eight studies were included in both quantitative (meta-analysis) and quantitative (descriptive) syntheses.

### 3.1. Study Characteristics

The eight studies included were carried out in the English language. Furthermore, the research examined was conducted across different countries and regions. The findings show that there are significant differences across the studies. In terms of the year, the studies included were conducted from 2017 to 2024. The region/country of the authors consisted of the USA, Sweden, the Netherlands, India, Iran, and Turkey [11,12,13,14,15,16,17,18]. The total sample size of this study was 1685, 12 was the sample size of the study with the lowest size [13] and 650 was the sample size of the study with the largest size [11]. The studies included involved prospective and retrospective research design methods, while the interventions were laparoscopic gastric bypass surgery and gastric bypass surgery.

Further, the studies focused on participants (women) of reproductive age, with a duration of infertility ranging from 9 months to 7 years. The results of all eight showed a decrease in the number of infertile women during the follow-up period. On the other hand, some studies showed a reduction in body mass index (BMI) during the study period [14,15,16,17]. Table 1 displays these results for each study across the rows, while the general features of the included studies are in columns.

### 3.2. An Assessment of the Quality of the Included Studies

The Newcastle–Ottawa Quality Assessment Scale (NOS) was used to assess the quality of the included studies (Table 2). For every numbered item in the selection and outcome categories, the studies received up to one star. However, comparability received a rating of up to two stars. Based on the assessment results, most of the studies were of a high quality with a low risk of bias [11,15,16,17]. However, a few studies showed a high risk of bias in the follow-up period for outcomes to occur and in the adequacy of follow-up of cohorts. A survey by Nilsson-Condori et al., 2020 and Snoek et al., 2024 showed a high risk of bias in regard to the follow-up period for an outcome to occur [12,14]. On the other hand, a study by Christinajoice et al., 2020 and Gunakan et al., 2020 showed a high risk of bias in regard to the adequacy of follow-up of cohort outcomes [12,14,15,17].

**Table 1:** The characteristics of the studies included in the systematic review.

Author	Region	Sample Size	Study Design	Intervention	Age	Duration of Infertility	Preoperative BMI	BMI at Conception	Inclusion	Outcome/Results
Menke et al., 2019 [11]	USA	650	Multicenter prospective cohort study	Sleeve gastrectomy	34	7 years	46.3	NA	Women who, prior to their preoperative or initial follow-up reproductive health examination, were between the ages of 18 and 44 and had no history of hormone replacement treatment, hysterectomy, or surgical or natural menopause.	When nulliparous women with a history of infertility before surgery were compared to those without, the former showed higher rates of early and postoperative pregnancy as well as a higher chance of engaging in unprotected sexual encounters.
Nilsson-Condori et al., 2020 [12]	Sweden	614	Retrospective analysis study	Laparoscopic gastric bypass surgery	25–34	18 months	41.9	NA	Women between the ages of 20 and 35, childless, proficient in Swedish, and approved for bariatric surgery.	After bariatric surgery, women report better body image and higher levels of self-esteem, which are crucial factors in their improved sexual performance.
Nilsson-Condori et al., 2019 [13]	Sweden	12	Prospective cohort study	Laparoscopic gastric bypass surgery	27.4	11 months	41.6	NA	Women without prior children who are between the ages of 20 and 35, speak Swedish, are eligible for bariatric surgery, and have been obese for more than five years with a BMI of over 40 or over 35 combined with one or more comorbidities.	It is generally agreed upon that better fertility after bariatric surgery is a positive and noteworthy consequence, even if the majority of obese young women do not seek the procedure for fertility-related reasons alone.
Snoek et al., 2024 [14]	The Netherlands	97	Retrospective cohort study	Gastric bypass surgery	Median age 29.8 (IQR 26.6–33.4)	9 months	43.6	29.8	At the Maasstad Hospital’s bariatric specialist center in Rotterdam, The Netherlands, only patients receiving pGB were included.	The study showed that pGB fetuses had significantly inferior fetal development parameters at 20 weeks of gestation and throughout the pregnancy compared to non-bariatric pregnancies. Lower birthweights were associated with pregnancies with pGB, increasing the risk of small-for-gestational-age births. There were no discernible differences in the mother’s pregnancy outcomes.
Christinajoice et al., 2020 [15]	India	45	Retrospective analysis study	Laparoscopic sleeve gastrectomy	Mean age 24.7 ± 10.2 years	3 years	48.5	31.8	A total of 45 patients (63.5%) out of the 71 females in the study have finished their three-year follow-up. Both hospital and outpatient records provided the data. Three groups of patients were studied: A, those with signs of polycystic ovarian disease (PCOD); B, those with primary infertility; and C, those who underwent bariatric surgery and became pregnant afterward.	Obesity and polycystic ovarian disease patients are closely associated with primary infertility. Women with polycystic ovarian disease who have bariatric surgery report significant improvements in menstrual irregularity and perinatal outcomes. Additionally, fertility greatly increases.
Khazraei et al., 2017 [16]	Iran	221	Retrospective study	Laparoscopic sleeve gastrectomy	18 to 63	5 years	48.94 ± 2.04	<27	Having given written informed consent and having a medically necessary LSG (BMI: 25–65).	A surgical treatment called laparoscopic sleeve gastrectomy is used to treat morbid obesity. It results in weight loss and the resolution of comorbidities. Young, obese, infertile women who want to get pregnant may find success with laparoscopic sleeve gastrectomy. Reducing body weight seems to improve irregular menstrual periods and raise the likelihood of conception. Two important aspects of managing infertility are the duration and magnitude of weight loss.
Gunakan et al., 2020 [17]	Turkey	23	Retrospective case-control study	Laparoscopic sleeve gastrectomy	32.4 ± 4.2	Group 1: ≤12 months; Group 2: >12 months	Before surgery and at the time of conception was 46.6 kg/m^2^ and 29.7 kg/m^2^	Before surgery and at the time of conception was 46.6 kg/m^2^ and 29.7 kg/m^2^	Women who became pregnant at the Keçiören Training and Research Hospital following a laparoscopic sleeve gastrectomy procedure performed for morbid obesity in 2017–2019.	Pregnancies that occur in the initial years following a sleeve gastrectomy appear to have comparable obstetric outcomes to those that occur later; however, this is still a contentious topic.
Ilyas et al., 2023 [18]	Turkey	23	Retrospective analysis study	Laparoscopic sleeve gastrectomy	31.26 ± 5.06 years	Five years	28.65 ± 3.14	NA	The study comprised 23 severely obese women, whose mean age was 31.26 ± 5.06 years (minimum 24, maximum 43), and whose mean marriage duration was 9.3478 ± 4.76 years (minimum 4, maximum 23). The women were followed up for five years after the study.	One significant surgical procedure for treating obesity and preventing its associated comorbidities is laparoscopic sleeve gastrectomy. Assisting obese infertile women with their weight loss and hormone balance through this procedure can increase the likelihood of pregnancy and live births.

PGP—pelvic girdle pain, BMI—body mass index, PCOS—polycystic ovarian syndrome, IVF—in-vitro fertilization, LSG—laparoscopic sleeve gastrectomy.

### 3.3. A Meta-Analysis of the Studies Outcome

**Figure 2:**
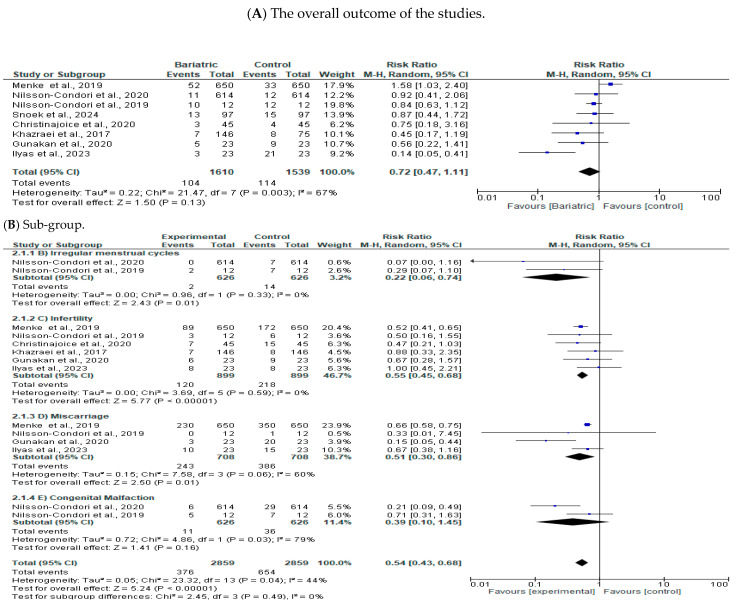
The effectiveness of bariatric surgery [11,12,13,14,15,16,17,18].

Figure 2A shows that the overall outcome of the studies used in the meta-analysis had moderately high heterogeneity I^2^ = 67%, Q^2^ = 0.22, an RR of 0.72, at a 95% CI (0.47, 1.11) (*p* = 0.0003). However, the overall heterogeneity of the included studies outcome was not statistically significant (*p* = 0.13). According to sub-group analysis, irregular menstrual cycles were found to have reduced significantly in favor of patients who underwent bariatric surgery (*p* = 0.01) with an RR of 0.22, at a 95% CI (0.06, 0.74) (Figure 2B (Irregular menstrual cycles)). With regards to infertility, the results indicate bariatric surgery reduced the level of infertility among the patients significantly (*p* = 0.00001) with an RR of 0.55, at a 95% CI (0.45, 0.68) (Figure 2B (Infertility)). Further, the results show bariatric surgery reduced the rate of miscarriage among patients (*p* = 0.01), with an RR of 0.51, at a 95% CI (0.30, 0.86) (Figure 2B (Miscarriage)). Moreover, bariatric surgery reduced the level of congenital malfunction, although the effect was not statistically significant (*p* = 0.16), with an RR of 0.39, at a 95% CI (0.10, 1.45) (Figure 2B (Congenital Malfaction)). The overall effect of bariatric surgery on treating infertility was found to be significantly effective (*p* = 0.0001), with an RR of 0.54, at a 95% CI (0.43, 0.68).

## 4. Discussion

Obesity is a significant factor influencing female fertility through various mechanisms, including disruption of the hypothalamic-pituitary-ovarian axis, which can lead to anovulation and infertility [10]. This systematic review and meta-analysis sought to establish the effectiveness of bariatric surgery in treating infertility among women. The study’s findings reveal that the overall effect of bariatric surgery on treating infertility was significantly practical (*p* = 0.0001) RR 0.54, at 95% CI (0.43, 0.68). Similar to this finding, a study by Chang et al. revealed that after bariatric surgery, the cases of infertility reported by the participants reduced significantly at each follow-up phase. The authors added that bariatric surgery helps infertile, overweight, and obese women lose weight, which improves ovulatory dysfunction and irregular menstruation while boosting spontaneous conception [19]. On the other hand, Kongkit et al. found in their study that after bariatric surgery, women who underwent the process reported giving birth safely at 36 weeks of gestation period. They further noted that within a year of bariatric surgery, spontaneous conception can occur because of decreased levels of androgens and increased levels of the follicular stimulating hormone and the sex hormone binding globulin [20]. This concurs with the finding in a study by Micic et al., who found bariatric surgery to have immediate benefits among infertile women. Compared to the group that did not undergo bariatric surgery, there was a decreased risk of developing preeclampsia and gestational diabetes mellitus after bariatric surgery. Another benefit noted in their study included a reduced risk of cesarean section and admission to the newborn critical care unit [21].

Moreover, despite the overall outcome of the studies used in the meta-analysis having moderately high heterogeneity I2 = 67%, Q2 = 0.22, RR 0.72, at 95% CI (0.47, 1.11) (*p* = 0.0003), the overall heterogeneity of the included studies’ outcomes was not statistically significant (*p* = 0.13). Irregular menstrual cycles were found to have reduced significantly in favor of patients who underwent bariatric surgery (*p* = 0.01), with an RR of 0.22, at a 95% CI (0.06, 0.74). These findings concur with the finding obtained in a study by Menke et al., which found that bariatric surgery significantly reduced the rate of irregular menstrual cycles among the participants surveyed by improving ovulatory dysfunction [11]. In addition, a study by Nilsson-Condori et al. also revealed that after bariatric surgery, women reported having regular menstrual cycles [12]. Nevertheless, in support of this finding, a study by Nilsson-Condori et al. found that obese women who underwent bariatric surgery saw improvements in their hormonal behavior over time, thus reducing the number of irregular menstrual cycles significantly over time [13].

Further, the finding of this study revealed that bariatric surgery reduced the rate of miscarriage among the treated women significantly (*p* = 0.01), with an RR of 0.51, at a 95% CI (0.30, 0.86). In line with this finding, a study by Snoek et al. found bariatric surgery to improve the level of fertility among obese women significantly [14]. In addition, a survey by Christinajoice et al. also found that after sleeve geriatric surgery was conducted among obese and polycystic ovarian disease patients who had primary infertility, their average BMI reduced significantly from 48.5 kg/m^2^ to 31.8 kg/m^2^. In contrast, the number of patients with successful childbirth increased over the 3-year follow-up period. They added that women who had polycystic ovarian disease and who received bariatric surgery reported a significant improvement in fertility due to the improvement in ovulatory dysfunction [15]. In support of this finding, a study by Khazraei et al. revealed that the duration and magnitude of weight loss are two essential aspects of managing infertility, which is achieved by undergoing bariatric surgery. They also noted that bariatric surgery helps overweight and obese individuals to lose weight, which in turn helps in the correction of irregular menstruation and ovulation dysfunction, thus reducing the rate of miscarriage [16].

Nevertheless, the forest plot diamond maker of the congenital malfunction fell on the treated group side as opposed to the control group; this indicates that bariatric surgery reduces congenital malfunction. However, the effect was not statistically significant (*p* = 0.16), with an RR of 0.39, at a 95% CI (0.10, 1.45), which can be attributed to the few studies included and the associated risk of bias. Aligned with this finding, a study by Gunakan et al. found that pregnancies that occurred in the initial years following a sleeve gastrectomy had successful outcomes compared to those that occurred later due to hormonal balancing after treatment [17]. A study by Ilyas et al. also noted that laparoscopic sleeve gastrectomy is one significant surgical procedure for treating congenital malfunction and preventing its associated comorbidities. They added that assisting obese infertile women with their weight loss and hormone balance can be very beneficial in increasing the likelihood of pregnancy and live births [18]. Based on this finding, it is plausible to say bariatric surgery is an effective method for treating infertile, overweight, and obese women.

This study had strengths as well as limitations. The strength of this systematic review was that it included a meta-analysis of the association between bariatric surgery and infertility, miscarriages, irregular menstrual cycles, and congenital anomalies. The use of a random-effects model accounted for the considerable amount of heterogeneity revealed among the included studies. Another strength was that the included studies were conducted from 2017 to 2024, which helps in obtaining up-to-date information regarding the impact of bariatric surgery among women of reproductive age and neonatal care practices in the healthcare setup. The main limitations of this study included the small number of studies and small sample sizes, which limits the generalizability of the study findings. Future studies should involve more studies with large sample sizes to generalize this finding in a broad context.

Further, most outcomes had high heterogeneity, which could be ascribed to differences in study designs and survey durations. Future studies should focus on studies with similar designs and approximately similar study durations in order to ensure conformity. Another limitation of this study was the low number of papers on the topic, which may hinder the rigorous exploration of the subjects under study. Future studies should include studies conducted for a long period to increase the number of studies on the topic in the assessment.

On the other hand, publication bias could not be evaluated per outcome due to the small number of pooled studies. Finally, this systematic review and meta-analysis focused on the overall impact of bariatric surgery on women of reproductive age. As such, future research needs to focus on the effectiveness of each type of bariatric surgery. This will significantly impact the way preconception care and counseling are provided. Further, this finding will inform guidelines used by healthcare insurance providers in the provision of bariatric surgery to improve the chances of conception among women with obesity.

## 5. Conclusions

This study found that bariatric surgery helps infertile women of reproductive age to lose weight, which improves ovulatory dysfunction and irregular menstruation while boosting spontaneous conception. On the other hand, this study noted that after bariatric surgery, spontaneous conception can occur because of a decreased rate of miscarriage, increased fertility, reduced levels of congenital malfunction, and the restoration of regular menstrual cycles. Therefore, this study highlights the need to offer adequate preconception care and counseling to women who are about to be pregnant, both before and after bariatric surgery. Further, based on the fact that this study focused on general bariatric surgery, future research should focus on specific types of bariatric surgery to establish the most effective type of bariatric surgery in treating infertility in women.

## Figures and Tables

**Table 2:** An assessment of the quality of the included studies.

Author	Selection				Comparability	Outcome			Overall Study Quality
	Representativeness of the Exposed Cohort	Selection of the Non-exposed Cohort	Ascertainment of Exposure	Demonstration that Outcome of Interest Was not Present at Start of Study	Comparability of Cohorts on the Basis of the Design or Analysis	Assessment of Outcome	Was Follow-Up Long Enough for Outcomes to Occur	Adequacy of Follow Up of Cohorts	
Menke et al., 2019 [11]	*	*	*	*	**	*	*	*	Excellent
Nilsson-Condori et al., 2020 [12]	*	*	*	*	*	*	-	-	Fair
Nilsson-Condori et al., 2019 [13]	*	*	*	*	*	*	*	*	Good
Snoek et al., 2024 [14]	*	*	*	*	*	*	-	-	Fair
Christinajoice et al., 2020 [15]	*	*	*	*	**	*	*	-	Good
Khazraei et al., 2017 [16]	*	*	*	*	**	*	*	*	Excellent
Gunakan et al., 2020 [17]	*	*	*	*	**	*	*	-	Good
Ilyas et al., 2023 [18]	*	*	*	*	**	*	*	*	Excellent

* shows the items present in the study. ** shows that the comparability was conducted appropriately.
